# Comparative transcripts profiling reveals new insight into molecular processes regulating lycopene accumulation in a sweet orange (*Citrus sinensis*) red-flesh mutant

**DOI:** 10.1186/1471-2164-10-540

**Published:** 2009-11-18

**Authors:** Qiang Xu, Keqin Yu, Andan Zhu, Junli Ye, Qing Liu, Jianchen Zhang, Xiuxin Deng

**Affiliations:** 1 National Key Laboratory of Crop Genetic Improvement, Huazhong Agricultural University, Wuhan 430070, Hubei, PR China

## Abstract

**Background:**

Interest in lycopene metabolism and regulation is growing rapidly because accumulative studies have suggested an important role for lycopene in human health promotion. However, little is known about the molecular processes regulating lycopene accumulation in fruits other than tomato so far.

**Results:**

On a spontaneous sweet orange bud mutant with abnormal lycopene accumulation in fruits and its wild type, comparative transcripts profiling was performed using Massively Parallel Signature Sequencing (MPSS). A total of 6,877,027 and 6,275,309 reliable signatures were obtained for the wild type (WT) and the mutant (MT), respectively. Interpretation of the MPSS signatures revealed that the total number of transcribed gene in MT is 18,106, larger than that in WT 17,670, suggesting that newly initiated transcription occurs in the MT. Further comparison of the transcripts abundance between MT and WT revealed that 3,738 genes show more than two fold expression difference, and 582 genes are up- or down-regulated at 0.05% significance level by more than three fold difference. Functional assignments of the differentially expressed genes indicated that 26 reliable metabolic pathways are altered in the mutant; the most noticeable ones are carotenoid biosynthesis, photosynthesis, and citrate cycle. These data suggest that enhanced photosynthesis and partial impairment of lycopene downstream flux are critical for the formation of lycopene accumulation trait in the mutant.

**Conclusion:**

This study provided a global picture of the gene expression changes in a sweet orange red-flesh mutant as compared to the wild type. Interpretation of the differentially expressed genes revealed new insight into the molecular processes regulating lycopene accumulation in the sweet orange red-flesh mutant.

## Background

Carotenoids are widely found in plant species and are responsible for the coloration of flowers and fruits to attract pollinators and seed-dispersing animals [1]. Carotenoids are essential components of human diets, and they have important roles in human health as antioxidants, vitamin A precursors and cancer-preventing effectors [2,3].

More than 700 naturally occurring carotenoids have been identified [4]. Of them, some carotenoids and their biosynthesis are well characterized; for example, α-carotene and β-carotene serve as important source of vitamin A [5,6]; and high levels of β-carotene accumulation in Cauliflower was mediated by an *Or *gene [7]. However, the majority of carotenoids, including lycopene, their biosynthesis and regulation are poorly understood. Lycopene provides the familiar red color to tomato fruits, and is the most potent antioxidant among carotenoids [8,9]. Interest in lycopene metabolism and regulation is growing rapidly because of the overwhelming reports on the role of lycopene in human health promotion, such as the prevention of a range of chronic diseases particularly the prostate cancer [10-12]. It has been shown in tomato fruit, the accumulation of lycopene is highly correlated with up-regulation of carotenogenic genes in the upstream of lycopene and down-regulation of lycopene cyclases in the downstream [13,14]. Biochemical and molecular studies in tomato mutants with different carotenoids accumulation have provided more in-depth insight into lycopene biosynthesis. One type is yellow-fruited tomato mutants with reduced lycopene content where two possibilities have been reported: one possibility is due to reduced expression of carotenogenic genes in the upstream of lycopene including *yellow-flesh *mutant due to dysfunction in phytoene synthase (*PSY*) [15], and *tangerine *mutant with a deletion mutation in carotenoid isomerase gene (*CRTISO*) [16]; the other possibility is due to up-regulation of lycopene downstream genes such as *Delta *mutant with increased expression of lycopene epsilon-cyclase gene (*LYCe*) [13]. For other types of mutants characterized with increased lycopene, the underlying mechanisms are more complicated. Ronen et al. (2000) analyzed an old-gold (*og*) mutant and found that lycopene accumulation is due to null mutation in the gene lycopene β-cyclase (*LYCb*) [17]. Three other mutants, named as *high pigment *(*hp*), are well-known for the accumulation of higher concentrations of lycopene in the fruit. Cloning of *HP-1 *gene showed that it encodes UV-damaged DNA binding protein (DDB1) [18]; *HP-2 *gene also encoded a light signaling regulator deetiolated1 (DET1) [19]; Whereas *hp-3 *mutation occurred in zeaxanthin epoxidase (*Zep*) gene, and caused abscisic acid deficiency [20]. Collectively, physiological, genetic and molecular studies of the mutants indicated that lycopene metabolism and regulation is complicated in tomato; however, in species other than tomato, there is limited information available on the mechanism of lycopene accumulation in fruits.

**Figure 1:**
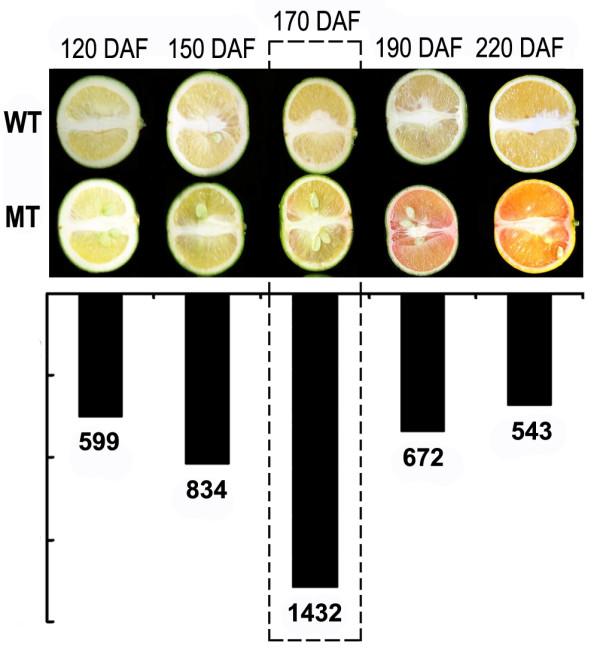
**Description of the materials used for MPSS analyses**. The picture shows the coloration changes of the fruit pulp in the mutant and wild type during fruit development (upper panel), and the differentially expressed genes at the five stages from our cDNA microarray data (lower panel, Liu et al., 2009). The five stages are 120, 150, 170, 190, and 220 days after flowering (DAF, X-axis). The fruit pulps of the mutant and wild type at 170 DAF, the stage with most significant transcriptional changes, were used for MPSS analyses in this study.

Sweet orange (*Citrus sinensis *[L.] Osbeck) is one of the most important fruit crops in the world. Sweet orange fruit is rich in carotenoids, and is reported with more than 115 species of carotenoids [21]. Carotenoid composition and content in sweet orange fruits have been extensively studied, and shown that lycopene is absent from common varieties [22-24]. So far, three sweet orange mutants with lycopene accumulation in the fruits were reported: Shara [25], Cara Cara [26] and the recently reported 'Hong Anliu' [27]. Most of the researches were on Cara Cara, including the analyses of carotenoid composition and content [23,28,29], and expression of the main carotenoid biosynthetic genes [30,31]. Alquezar et al. (2008) found that the altered carotenoid composition in Cara Cara may conduct to a positive feedback regulatory mechanism of carotenoid biosynthesis during fruit development and maturation; and not only carotenogenic genes but also the isoprenoid genes were altered transcriptionally in the mutant. The other red-flesh mutant 'Hong Anliu' was discovered in China as a bud mutation of 'Anliu' sweet orange; and we found that lycopene in this mutant is 1000-fold higher than that in comparable wild type fruits, and in juice sacs the lycopene accumulation was coincided with increased expression of upstream carotenogenic genes and reduced expression of downstream genes. Interestingly, this bud mutation also caused high sugar and low acid in the mutant fruits [27]. Molecular evaluation on DNA level using SSR with 80 primers and a number of AFLP markers produced no polymorphism between them, indicating an isogenic background between them (data unpublished). Thereafter, we used suppression subtraction hybridization (SSH) combined with cDNA microarray technique to investigate the molecular basis of the bud mutation. A total of 267 differentially expressed genes were detected [32]. Interestingly, 95% of the 267 genes showed differential expression at 170 days after flower (DAF), indicating that the 170 DAF is a critical stage for the transcriptional regulation of the mutant trait formation (Figure 1). Taken together, our previous research by SSH technology has provided important clues for understanding the formation of mutation trait in 'Hong Anliu', however, the transcriptional information from SSH, especially for the gene expressed in low levels, are rather limited. Further researches of the global transcriptional analyses are needed.

Massively parallel signature sequencing technology (MPSS) is a sequence-based method recently developed that can be used for measuring gene expression [33,34]. The MPSS method is "global" in that it can identify a nearly complete inventory of transcripts in a given sample. For plant species that lack whole genome sequence information such as citrus, MPSS can provide a broader view of the transcriptome than microarray analyses which includes known sequences [35]. The MPSS method has been used for various transcriptional studies in plants [36-38]. Two interesting cases in *Arabidopsis *mutant-wildtype pairs have suggested that MPSS method is robust in identifying mutation responsive genes [39,40].

In this study, a genome-wide gene expression study was carried out between the red-flesh mutant 'Hong Anliu' and its wild type 'Anliu' sweet orange using MPSS method, A total of 20,178 genes were analyzed, of which 2,936 genes in sense and 802 genes in antisense showed a two-fold or greater expression difference between the mutant and wild type, and 452 genes in sense and 130 genes in antisense are differentially expressed at 0.05% significance level. The results demonstrated that some genes are newly transcribed in the mutant. Our results also provided a large number of genes previously not known to be involved in the mutation trait formation. Interpretation of the data built up links between new information herein and our previous fragmentary knowledge, and provided new insight into the molecular processes regulating lycopene accumulation in the mutant fruits.

## Results

### MPSS signature abundance and distribution

MPSS libraries were constructed using RNA extracted from sweet orange fruit pulps at 170 DAF stage for wild type (WT) 'Anliu' sweet orange and its red-flesh mutant (MT) 'Hong Anliu'. A total of 6,983,578 and 6,468,017 successful sequences were produced for WT and MT, respectively. The sequence sets were filtered to remove low quality sequences containing ambiguous nucleotides, adaptor sequence and below 3 transcripts per million (TPM) in both libraries, resulting in 6,877,027 reliable signatures for WT and 6,275,309 reliable signatures for MT (the reliable sequence was termed as 'signature' hereafter). From the reliable signature sets, 144,810 and 156,582 distinct signatures were observed for WT and MT, respectively (Table 1). Correlation efficient of the MPSS data between WT and MT revealed high repeatability with r = 0.88. The saturation evaluations showed that with the increase of total sequence number (sequencing depth), the number of new distinct signature decreased markedly; and particularly the new distinct signature with frequency >1 decreased to 0 when the total sequences reached 6 million (see additional file 1). This indicated that the library size is saturated and contained enough signature information for gene expression analyses.

**Table 1:** Summary statistics of MPSS signatures in the red-flesh mutant 'Hong Anliu' sweet orange (MT) and its wild type 'Anliu' sweet orange (WT)

	WT		MT	
Total Sequence Collected	6983578		6468017	
Low Quality Signatures	106551	1.53%	192708	2.98%
Reliable Signatuers	6877027	98.47%	6275309	97.02%
Distinct Signatures	144810	2.07%	156582	2.42%

The distribution of signature abundance was quite similar between MT and WT (see additional file 2). Three signatures in MT and four signatures in WT were expressed at high abundance more than 1% (>10,000 TPM). While with the decrease of abundance, the number of signatures increased dramatically. 60% of the total signatures in MT and 56% of the total signatures in WT were at abundance less than 0.0001%. Moreover, about 99% of the total signatures in both libraries was below 0.001% abundance (<10 TPM), suggesting that genes with low transcripts are abundant in both libraries.

### Differential expression of MPSS signatures between mutant and wild type

The frequency of signature was regarded as relative expression level of each transcript in MT and WT libraries. Comparative analyses of the frequency of signature between MT and WT revealed that the expression ratio (MT/WT) varied greatly from 0.008 to 828. Of the common signatures in both libraries, 29,602 signatures showed a two-fold or greater (ratio>2 or <0.5) expression difference between MT and WT (see additional file 3), and are regarded as differentially expressed transcripts according to the criteria defined by Meyers et al. (2004b) [34].

**Figure 2:**
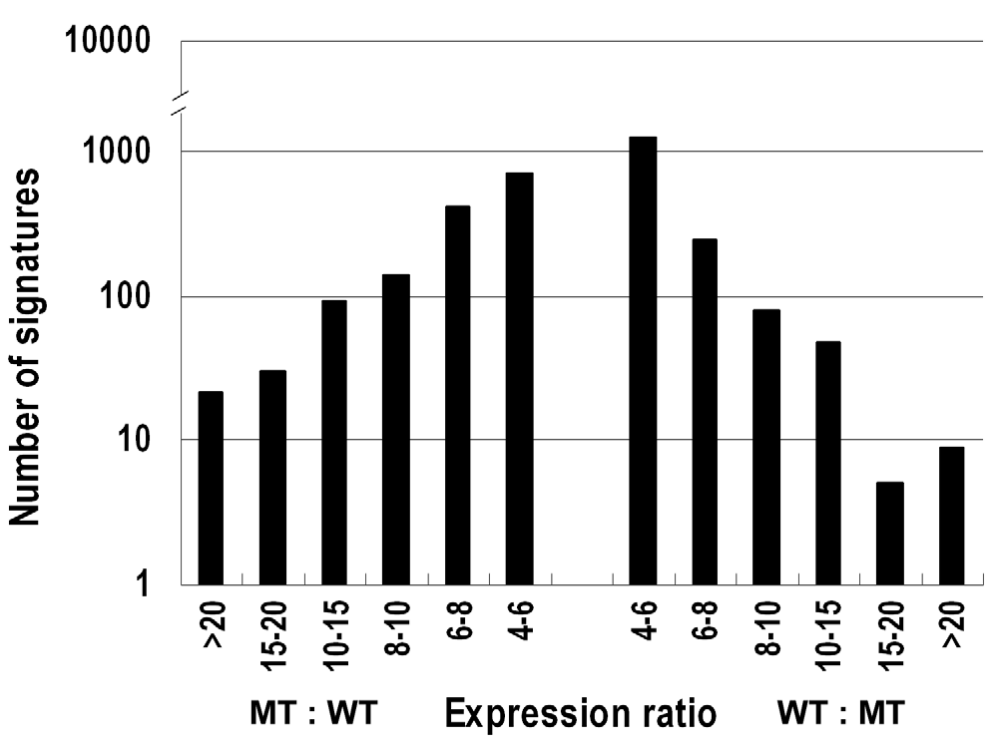
**Comparison of expression of the MPSS signatures between the mutant and the wild type**. The expression ratios compare the abundance of each signature between mutant and wild type. Columns denote the number of signatures with an expression ratio within the stated range.

Signature frequency was also compared statistically between the two libraries using Z-score method according to Kal et al. (1999) [41], which use *p*-value as statistical significance level. This method revealed that 3,036 signatures were significantly different at p < 0.05, at the same time their expression ratio were greater than 4 or less than 0.25 (Figure 2). Of these 3,036 signatures, 707 (23%) were significant at p < 0.01 with their expression ratio >6 or <0.17.

### Identification of mutation-responsive genes

To link the signatures to known genes from sweet orange, a unigene dataset comprised 26,826 contigs and 73,607 singletons was used for MPSS signature mapping analyses. All the sequences were used to produce *in silico *signatures in both sense and antisense directions. A total of 176,549 and 171,355 *in silico *signatures were identified in sense and antisense respectively. The produced *in silico *signatures stored in the reference signature database, through which the expressed MPSS signatures were mapped on the corresponding EST contigs and singletons based on matches between MPSS signatures and *in silico *signatures. The results showed that a total of 18,106 genes (16,005 in sense and 2,101 in antisense) are expressed in MT (see additional file 4); and 17,670 genes (15,697 in sense and 1,973 in antisense) are expressed in WT (see additional file 5).

**Figure 3:**
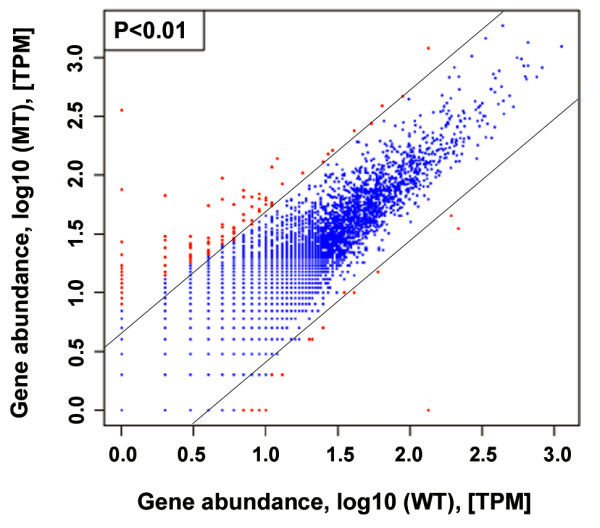
**Comparison of unigenes expression between the mutant and the wild type**. The abundance of each gene was normalized as transcripts per million (TPM). Up- or down-regulated genes, as defined at 0.01% significance level, are shown in red, and genes not differentially expressed between the mutant and the wild type are shown in blue.

The expression level of each gene in MT and WT was measured by the normalized number of the frequency of MPSS signature (occur as TPM) to look for genes showing significant changes between the two samples. A general picture of the gene expression was plotted for the MT versus that of WT (Figure 3). A total of 2,936 genes in sense and 802 genes in antisense showed a two-fold or greater (ratio>2 or <0.5) expression difference between MT and WT. Based on Z-score statistical analyses at 0.05% significance level, 452 genes in sense and 130 genes in antisense were up- and down-regulated (see additional file 6); and all these 582 differentially expressed genes showed a significant induction or repression by more three-fold. The significantly differential expressed genes can be directly linked to carotenoid biosynthesis (e.g. TC5, *LCYb *gene); or consistent with our recently published microarray data (e.g. TC2886, malate synthase; Liu et al., 2009); or in line with our proteomic data (e.g. stress response gene CN187632; Data unpublished); but most of them were newly observed in this study, such as ABA responsive genes (TC23639; TC7532), gibberellin related genes (TC22276; TC23691; TC18809), terpenoid biosynthesis related genes. Moreover, a number of transcription factors including MYB and NAC were observed (detail in Table 2).

**Table 2:** List of the important KEGG pathways more than 3 differentially expressed genes affiliated

KEGG^**a **^pathway	**Genes**^**b**^	Gene ID	Best E-value
Apoptosis	5	CN183092, TC22936, TC25357, TC3430, TC81	4.00E-025
Biosynthesis of steroids	5	EY708186, EY737600, TC13179, TC14003, TC8568	1.00E-145
Calcium signaling pathway	4	TC10446, TC2776, TC6871, TC41	1.00E-021
Carotenoid biosynthesis-General	4	TC14843, TC17929, TC5, TC5834	0
Cell cycle	4	TC410, TC15757, TC25101, TC5900	1.00E-030
Citrate cycle (TCA cycle)	3	EY670548, TC22243, TC623	1.00E-166
Cysteine metabolism	4	CF835920, DN620599, TC12093, TC5490	1.00E-073
Diterpenoid biosynthesis	4	TC14843, TC17929, TC22276, TC23691	8.00E-043
Fatty acid biosynthesis	7	CX074436, EY700768, CV713900, EY748567, TC10790, TC13490, TC9950	1.00E-133
Flavonoid biosynthesis	4	BQ622999, TC18809, TC23691, TC24546	1.00E-063
Folate biosynthesis	3	TC13190, TC25006, TC9636	1.00E-105
Fructose and mannose metabolism	3	TC3179, TC599, TC8180	1.00E-144
Glycerophospholipid metabolism	3	CX676461, TC13437, TC1366	3.00E-042
Glycolysis/Gluconeogenesis	5	TC10790, TC13490, TC5567, TC599, TC4947	1.00E-144
Limonene and pinene degradation	4	TC12405, TC14251, TC15293, TC20473	1.00E-125
MAPK signaling pathway	4	TC10975, TC11537, TC14581, TC23673	7.00E-075
Metabolism by cytochrome P450	3	TC16950, TC25526, TC25649	2.00E-052
Nicotinate and nicotinamide metabolism	4	DN620930, TC12023, TC19292, TC23834	2.00E-063
Oxidative phosphorylation	12	CX047553, DN621543, EY666061, TC10047, TC11705, TC16046, TC165, TC2473, TC2776, TC367, TC9593, TC9609	0
Photosynthesis	5	DY305711, EY675075, TC15280, TC17840, TC225	1.00E-124
Proteasome	3	TC5683, TC15808, TC4307	1.00E-134
Protein export	3	EY655396, EY667540, TC3601	3.00E-085
Pyruvate metabolism	8	928, CV713900, EY670548, TC13490, TC13985, TC2886, TC4947, TC623	1.00E-166
Ribosome	11	EY678532, EY755319, EY756332, TC13063, TC17838, TC18427, TC20162, TC21064, TC25973, TC7029, TC8912	8.00E-095
Starch and sucrose metabolism	4	TC12327, TC6279, TC7121, TC7710	0
Ubiquitin mediated proteolysis	12	EY692161, EY720797, TC11061, TC11537, TC11678, TC11708, TC11720, TC15530, TC18081, TC24614, TC458, TC8922	1.00E-116

^a^KEGG = Kyoto Encyclopedia of Genes and Genomes.

^b^The differentially expressed genes are significant at 0.05 level between the mutant and wild type

### Functional classification of mutation-responsive genes

**Figure 4:**
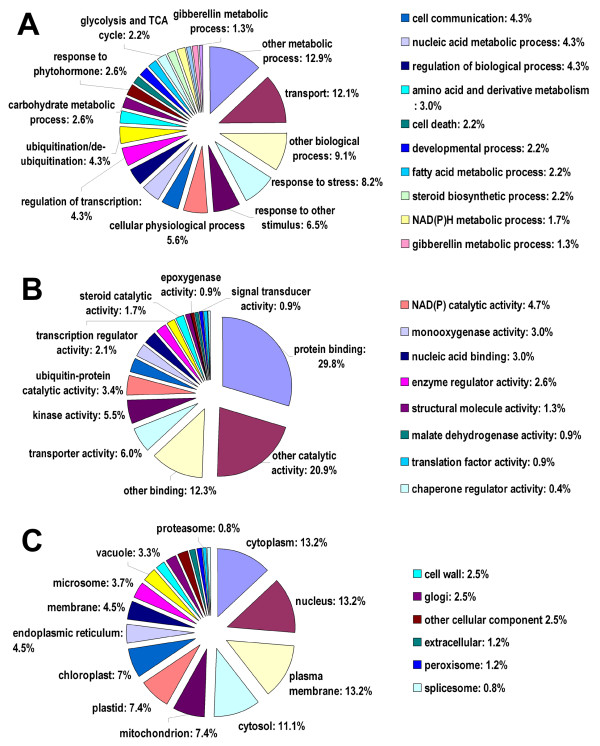
**Functional categorization of the genes with significant transcriptional changes between the mutant and the wild type**. The genes were categorized based on Gene Ontology (GO) annotation and the proportion of each category is displayed based on: Biological process (A); Molecular function (B); Cellular component (C).

To evaluate the potential functions of genes that showed significant transcriptional changes between the MT and WT, gene ontology (GO) categories were assigned to the significant 582 genes using Annot8r program. The categorization of mutation-responsive genes according to the cellular component, molecular function, and biological process are shown in Figure 4. Based on molecular function, the genes were finally classified into 18 categories, as shown in Figure 4B; The most over-represented GO terms (high p value and at least 3 genes were associated to the term) concerned with protein binding, transporter activity, NADH(P) catalytic activity, ubiquitin-related activity, protelysis activity and transcription regulator. Categories based on biological processes revealed that the mutation responsive genes were related to 23 biological processes, including transport, response to stress, regulation, ubiquitination, steroid biosynthesis, fatty acid metabolism, glycolysis and TCA cycle and etc (Figure 4A). Moreover, GO representations from this study were compared with that based on all the unigenes from sweet orange in TIGR gene index database [42]; the results revealed that the striking differences lies in the high percentage of plastid and chloroplast for cellular component, and overpresentation of photosynthesis, citrate cycle and ubiquitination for biological process in this study.

The biological interpretation of the significant mutation responsive genes was further completed using KEGG pathway analyses. A total of 26 different metabolic pathways were found with more than 3 genes affiliated, of which some were consistent with biological processes already revealed by GO analyses. The most represented pathways were listed in Table 2. Of these, some were related with mutation trait formation based on previous knowledge, including carotenoid biosynthesis (4 enzymes represented), citrate cycle (3), diterpenoid biosysnthesis (4), fatty acid metabolism (7), glycolysis (5), oxidative phosphorylation (12), photosynthesis (5), pyruvate metabolism (8), starch and sucrose metabolism (4), steroids biosynthesis (5), and ubiquitin mediated proteolysis (12).

### Verification of the mutant trait formation related genes

**Figure 5:**
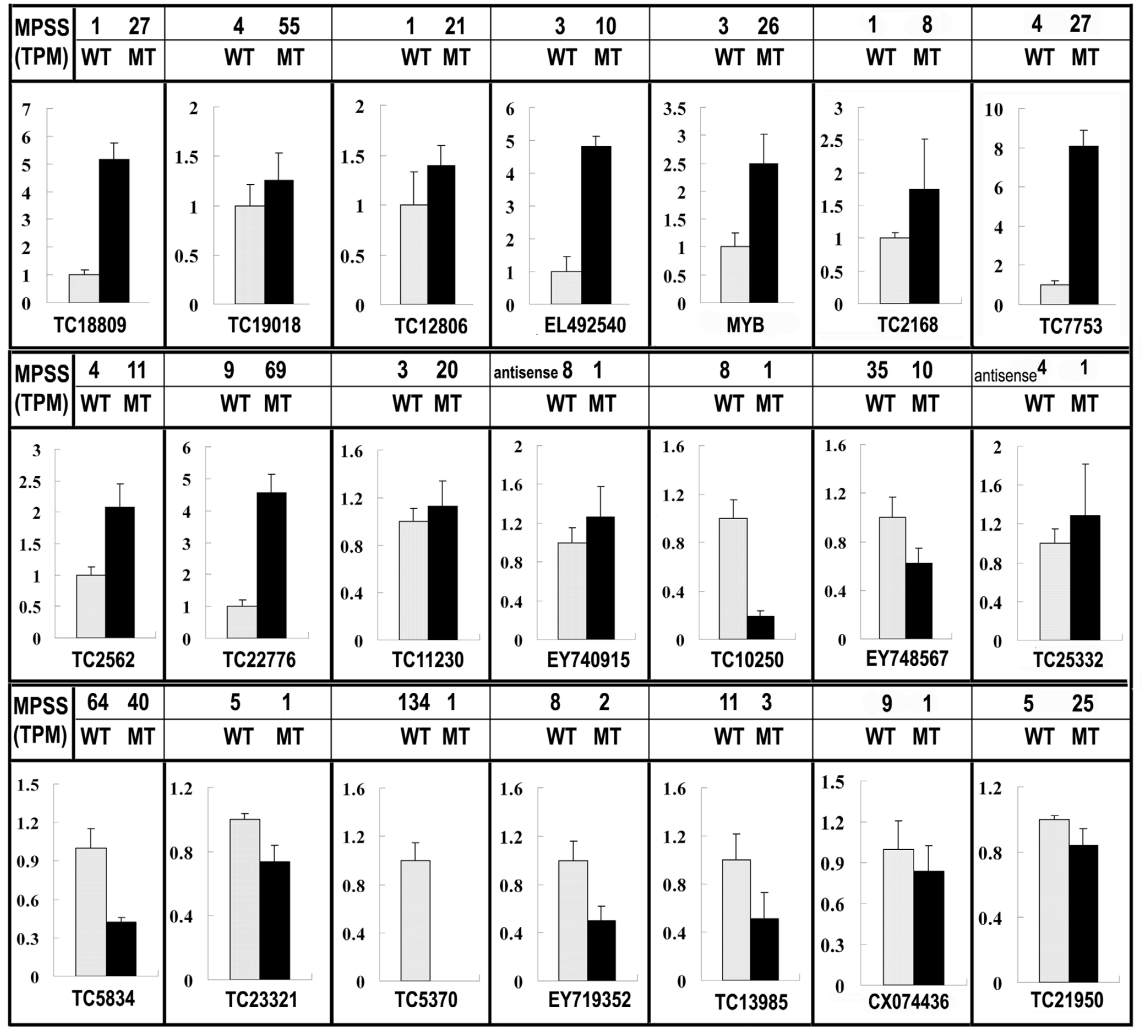
**Real-time quantitative RT-PCR confirmation of the differentially expressed genes between the wild type (grey columns) and the mutant (black columns)**. Columns and bars represent the means and standard error (n = 3) respectively. The transcript abundance from MPSS data was added on the top of each gene. TPM, transcripts per million.

**Figure 6:**
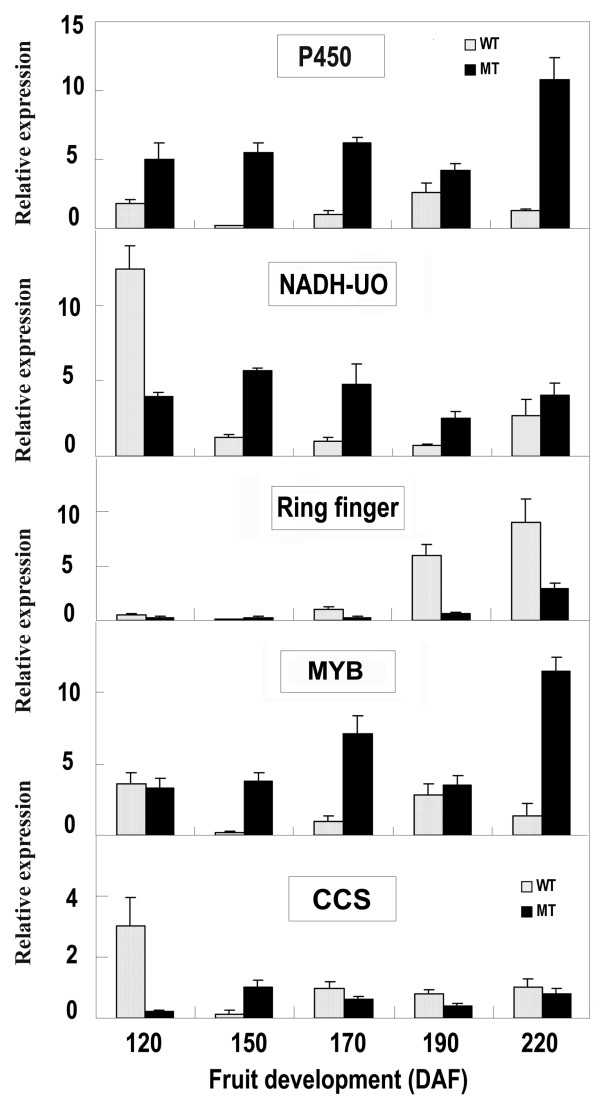
**Transcript level of 5 selected genes at different stages of fruit development in the mutant (black columns) and wild type (grey columns)**. The relative expression levels (Y-axis) were analyzed by real-time PCR. Columns and bars represent the means and standard error (n = 3), respectively. The genes are cytochrome P450 (designated as P450 in the picture), NADH-ubiquinone oxidoreductase (NADH-UO), Ring finger gene (Ring finger), MYB transcription factor (MYB), and Capsanthin/capsorubin synthase gene (CCS).

Transcriptional regulation revealed by MPSS data was confirmed in a biologically independent experiment using quantitative RT-PCR. A total of 25 genes, including 24 significantly differentially expressed genes and one gene of no differential expression, were chosen to design gene-specific primers (see additional file 7). The transcript abundance patterns of the MT and WT were compared with MPSS data. Results showed that for 20 of the 24 genes, qRT-PCR revealed the same expression tendency as the MPSS data, despite some quantitative differences in expression level. Figure 5 showed 21 genes (10 for induced, 9 for repressed, and 2 for antisense genes) expression levels between MT and WT. For example, the photosynthesis-related gene TC7753 showed 8.1 times up-regulation in MT than in WT as analyzed by qRT-PCR, consistent with MPSS data that the gene expression in MT was 6.8-folds higher than the WT. Furthermore, the expression profile of five genes including MYB transcription factor, capsanthin synthase (*CCS*, a gene functions downstream of lycopene), and three other newly detected genes with significant transcriptional changes (TC18809, TC1908, and TC10250) were analyzed at five stages during fruit development between the mutant and wild type (Figure 6). As expected, the *CCS *gene is down-regulated in MT at 170 DAF stage, indicated that partial impairment of lycopene downstream flux can be caused by down-regulation of *CCS *gene. It is noticeable that the expression level of the MYB gene in MT is 7.1 fold higher than that in WT, correlating well with the MPSS data showing 8.7 fold expression difference at 170 DAF stage.

### Measurement of chlorophyll content and photosynthesis activity

**Figure 7:**
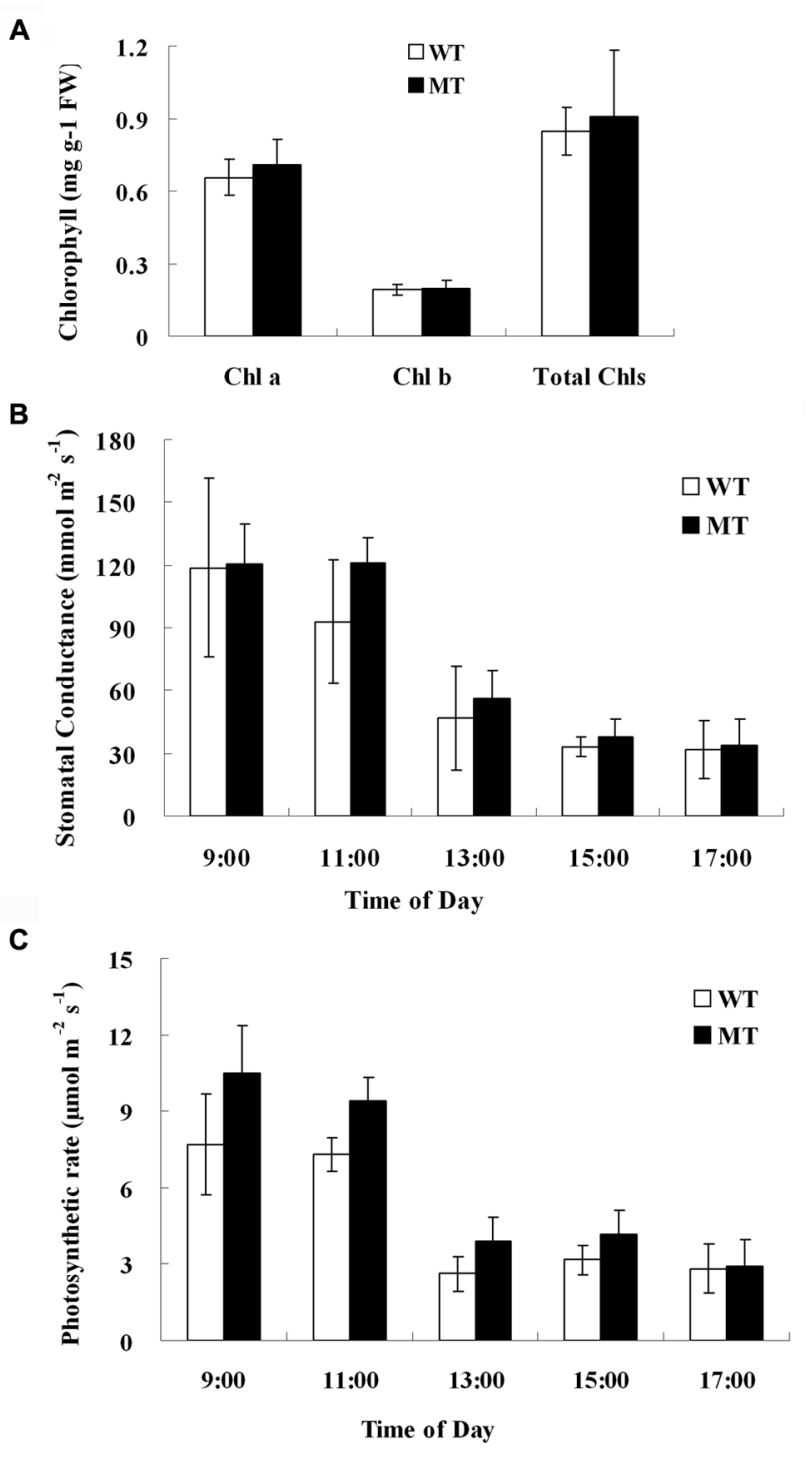
**Chlorophyll content and photosynthesis activity differences in the mutant and wild type**. A: chlorophyll content in leaves of mutant and wild type; B: stomatal conductance measured at five time points of the day (X-axis); C: Photosynthetic rate differences between the mutant and wild type. The stomatal conductance and photosynthetic rate were measured using a TPS-1 portable photosynthesis system. Wild type was denoted as WT (white column) and mutant as MT (black column). Three independent plants for each genotype and four leaves from each plant were measured; the average values and standard deviation were displayed.

The chlorophyll content and photosynthesis activity were measured in leaves of three different trees each for WT and MT. Measurement of chlorophyll content did not reveal significant difference between WT and MT, though the total chlorophyll content in MT is slightly higher than that in WT (Figure 7A). The photosynthetic rate and stomatal conductance were measured at 5 time points of the day. Results showed that the stomatal conductance in MT is higher than that in WT at all the stages, with significant difference at 11:00 am in the morning (Figure 7B). The highest photosynthetic rate was measured at 9:00 am in the morning, and the difference between WT and MT was significant at all the 5 time points except at 17:00 pm in the afternoon (Figure 7C). All this physiological data revealed that photosynthesis, as one of the important biological processes, was enhanced in the MT.

## Disscussion

MPSS, like expressed sequence tags (ESTs) and serial analyses of gene expression (SAGE), is a tag-based method recently developed that can be used for quantitative measurements of gene expression when combined with genomic sequence or unigene dataset [33,43]. Comparing with ESTs and SAGE technologies, MPSS has its advantages that it can provide a more thorough and scientific representation of the absolute transcript population, and is more sensitive to genes expressed at low levels due to deep sequencing with the resultant dataset containing more than 6 million tags for each sample in this study (also discussed in [38]). When compare with cDNA microarray technology that requires previous knowledge of genes, the limitations to detect unknown genes was not encountered in MPSS [32,39]. Another difference between MPSS and microarray is that statistical analyses in microarray is based on biological replicates; while statistical analyses of MPSS is usually based on enough sequences with more than 1 million tags in a given sample [35,38,43]. The MPSS technology has become popular in transcriptional expression studies [36]. From the results of this study, it is easy to notice that MPSS analyses not only highlight some genes and biological processes already revealed by our microarray and proteomic data, but also reveal large amount of genes which are possibly involved in the formation of mutation trait. The data consistency from multiple approaches assures that the MPSS data produced in this study is reliable.

### Identification of key genes and metabolism pathways involved in the formation of lycopene accumulation trait in the sweet orange mutant

**Figure 8:**
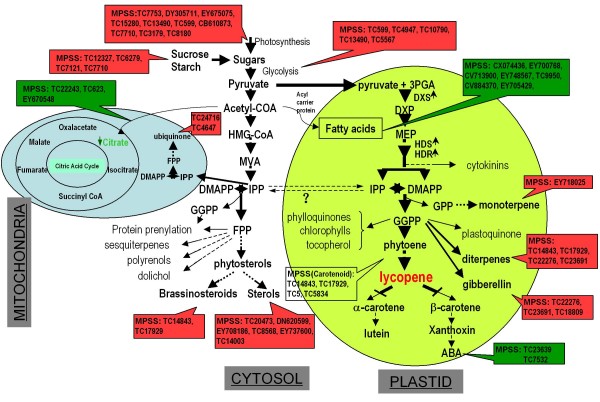
**Overview of major metabolic pathways involved in the mutant trait formation, as suggested by the interpretation of the GO and MPSS data**. Part of the model was modified from Figure 1 of Rodriguez-Concepcion (2006) [62]. The box background in red indicates up-regulated genes, and green indicates down-regulated genes. The genes are designated as TC numbers from TIGR gene index database, and the detailed gene information can be viewed in additional file 6. The TC numbers for *LYCb *and *CCS *genes were TC5 and TC5834 respectively. The abbreviations are as follows: **3PGA**, glyceraldehyde 3-phosphate; **ABA**, abscisic acid; **DMAPP**, dimethylallyl diphosphate; **DXP**, deoxyxylulose 5-phosphate; **DXS**, deoxyxylulose 5-phosphate synthase; **FPP**, farnesyl diphosphate; **GPP**, geranyl diphosphate; **GGPP**, geranylgeranyl diphosphate; **HDR**, hydroxymethylbutenyl 4-diphosphate reductase; **HDS**, hydroxymethylbutenyl 4-diphosphate synthase; **HMG-COA**, hydroxymethylglutaryl coenzyme A; **IPP**, isopentenyl diphosphate; **MEP**, methylerythritol 4-phosphate; **MVA**, mevalonic acid.

In this study, we used MPSS method to monitor the global transcriptional changes in the MT comparing with WT, and identified 582 differentially expressed genes at 0.05% significance level that were induced or repressed by more than three fold in the mutant. A number of new genes possibly related with lycopene accumulation were found in this study. Functional category analyses revealed that a number of important pathways may work collaboratively in shaping the red-flesh trait in the mutant (Figure 8).

The first noticeable pathway is the isoprenoid and carotenoid biosynthesis pathways which exist in plastid. Four differential genes are involved in the carotenoid metabolism pathway, including TC5 gene (encoding lycopene β-cyclase, *LYCb*), TC5834 gene (encoding capsanthin synthase, *CCS*), and two different abscisic acid hydroxylases (hydroxylase 2 and 4), encoded by TC14843 and TC17929 genes (Figrue 8). The transcription level of *LYCb *gene detected by MPSS in mutant was 6 times lower than that in wild type, consistent with our previous data by qRT-PCR [27]. The LYCb was believed to be a rate-limiting enzyme in the conversion of lycopene to cyclic carotenes [44]. The *CCS *gene is also significantly down-regulated in the mutant at 170 DAF (Figure 6). The CCS is an enzyme with a similar action mechanism to LYCb; and low transcript level of CCS is possibly responsible for the accumulation of lycopene in red grapefruits (*Citrus paradisi*) [45]. Based on these data, it can be speculated that the down-regulation of lycopene downstream genes *LYCb *and *CCS*, in combination with up-regulation of lycopene upstream genes (i.g. *PSY*, our previous data [27]), will result in the accumulation of lycopene in the sweet orange mutant. It is interesting that the biosynthesis of abcisic acid (ABA) was affected in the mutant. Two genes involved in ABA biosynthesis were down-regulated, fitting well with our observation that ABA content in MT is lower than that in WT (data unpublished). The decreased biosynthesis of ABA in MT is plausible due to the decreased supply of ABA precursor, xanthoxin, which is downstream of lycopene and could be significantly affected by down regulation of *LYCb *gene. This could also be evidenced from another sweet orange mutant 'Cara Cara' with high lycopene accumulation that the pulp of the mutant at color-breaker stage only contained 23% of the ABA content of its normal counterpart [31]. A recent study in tomato *hp3 *mutant also showed that ABA is deficient in the mutant with higher lycopene content [20].

The second group of significant mutation-responsive pathways includes photosynthesis, glycolysis, citrate cycle (TCA cycle) and fatty acid metabolism. Pathways in this group showed a complicated pattern. For photosynthesis, 10 genes were involved as revealed from GO and KEGG analyses; all these genes exhibited up-regulation pattern in MT fruits (Figure 8). The induction of genes associated with photosynthesis is consistent with the GO results that large number of genes are located in chloroplast; and is in line with our gas chromatographic analyses that the sugars in juice sacs is higher in MT than in WT [27]. Moreover, our in situ analysis of the photosynthetic rate confirmed that photosynthesis in MT is stronger than that in WT (Figure 7). Another case in tomato *hp-2 *mutant which manifested with increased carotenoids, primarily lycopene, showed that genes involved in photosynthesis are consistently up-regulated throughout fruit ripening [46]; and by contrast sugar deficiency delayed lycopene accumulation in tomato [47]. All these findings suggest the importance role of photosynthesis and sugar in the accumulation of lycopene. Similar to photosynthesis, genes involved in glycolysis showed an up-regulated pattern. The glycolysis provides substrates pyruvate for the methylerythritol phosphate (MEP) pathway. Meanwhile, our data showed three important genes participated in MEP pathway (*DXS*, *HDS *and *HDR*) were also up-regulated (see additional file 8). Therefore, the increased MEP biosynthesis led to the enhanced biosynthesis of carotenoids, and meanwhile resulted in the dramatical increment of lycopene due to the partial impairment of downstream flux caused by down-regulation of *LYCb *and *CCS *gene as discussed in the previous paragraph (Figure 8). This provides a molecular scenario for the formation of lycopene accumulation trait in the mutant red-flesh fruits. In the other metabolic flux of pyruvate which happens in cytosol, acetyl-COA is produced for the mevalonic acid (MVA) pathway. And the two branch flux of acetyl-COA were inhibited. One is the TCA cycle; three genes, isocitrate dehydrogenase (TC22243), malate dehydrogenase (TC623), and importin alpha-4 gene (EY670548), were down-regulated. These MPSS data correlate well with our previous data that the citric acid in the mutant is significantly lower (nearly one quarter) than that in wild type [27]. The other branch downstream of acetyl-COA is fatty acid metabolism; all the genes involved in this pathway were down-regulated.

The third group of metabolite pathways is downstream of MVA pathway. This group is relatively simple. Two genes involved in the ubiquinone biosynthesis were up-regulated. Six genes related to sterol biosynthesis and two to brassionsteroids biosynthesis also showed an up-regulated pattern in the mutant.

When our results on red-flesh orange mutant is compare with that on tomato mutants with abnormal accumulation of lycopene, the molecular mechanism for lycopene accumulation is different. For example, lycopene accumulation in tomato *old-gold *mutant is due to null mutation in *LYCb *gene [17]; and *high-pigment 3 *mutant is due to mutation on *Zep *gene [20]; While tomato *high-pigment 1 *and *high-pigment 2 *mutation happened on light responsive genes [18,19]. In this study, by using a pleiotropic mutant with high sugar, low acid and abnormal accumulation of lycopene, extensive molecular pathways including isoprenoid biosynthesis, carotenoid biosynthesis and photosynthesis were affected in the mutant, and enhanced photosynthesis and the partial impairment of lycopene downstream flux caused by down-regulation of *LYCb *and *CCS *genes are critical for the formation of lycopene accumulation trait in the sweet orange mutant.

### The transcriptional regulation of carotenoid metabolism in sweet orange mutant

The alternation of carotenoid composition and concentration can be achieved through biosynthesis and post-biosynthesis activities including transport, storage and degradation [2]. These activities could be regulated on transcriptional and posttranscriptional levels. In our study, high percentage of antisense transcripts, i.e. 2,101 of 18,106 genes in MT and 1,973 of 17,670 genes in WT, were observed, possibly indicating post-transcriptional regulations existed in sweet orange fruit development. In contrast, the MPSS data provided much more information for the regulation of these activities on transcription level.

A noticeable result is that considerable amount of genes, 8.6% of the total annotated genes, were involved in regulation of biological process or transcription (Figure 4). The induction of regulatory genes of transcription correlates well with the increased overall transcription in MT. The total number of transcribed genes in the mutant was 18,106, more than that in wild type 17,670, suggesting that newly initiated transcription occurs in the mutant. Moreover, analyses of the genes with transcription changes > 2 fold showed that up-regulated genes constitute 62.7% of the total changed genes. It is noticeable that *MYB *gene, a transcription factor (TF), showed a significant transcription changes in the mutant as revealed by MPSS data. And qRT-PCR analysis confirmed that the differential expression of *MYB *gene was significant at 150, 170 and 220 DAF stages, suggesting that *MYB *gene functions in extensive stages during fruit development. *MYB *genes are a superfamily of transcription factors that control many biological processes; and have also been highlighted for their regulation in pigment accumulation, primarily in anthocyanin biosynthesis [48-51]. While, to the author's knowledge, it is still unknown whether *MYB *genes are regulating carotenoid biosynthesis; so this study provided an important gene that possibly regulate lycopene accumulation in the orange fruits.

Genes possibly involved in the post-biosynthesis activities were also highlighted with significant transcriptional changes. An impressive category is transporter gene, which constitutes 12.1% of the total annotated genes. One gene EY748567 shows high homology with sugar transporter gene (67% similarity), suggesting that sugar translocation is active in 170 DAF stage. This is consistent with the above mentioned result that photosynthesis are active, and thus the produced sugar has to be transported to juice sac through phloem in citrus [52]. Our physiological data also showed that the stage 170 DAF is also the most important stage for sugar accumulation (data unpublished). Another remarkable group possibly involved in post-biosynthesis activities is ubiquitination or proteolysis related genes. Twelve genes were categorized into ubiquitin mediated proteolysis (Figure 4), indicating that protein degradation may play important role in maintaining certain important gene at a constant steady-state level, as proposed by Welsch et al. (2007) who found a ring finger protein involved in degradation processes and is a stable element in the carotenogenesis of Arabidopsis leaves [53]. In this study, the ring-finger gene (TC10250) was mainly expressed in mature stages of the fruits, possibly indicating that ring finger gene has a similar function as a stable element in carotenogenesis during orange fruit development.

## Conclusion

Our study provides a global picture of the gene expression changes in a sweet orange red-flesh mutant comparing with wild type. The interpretation of the MPSS data uncovered a large number of genes which were previously not known to be involved in the mutation trait formation. Functional categorization of the differentially expressed genes showed that a number of important pathways, including isoprenoid biosynthesis, carotenoid biosynthesis, photosynthesis, citrate cycle (TCA) and some post-biosynthesis activities such as transporter and degradation, cross communicated and worked collaboratively in shaping the red-flesh trait of the mutant. This study provided new insight that enhanced photosynthesis and the partial impairment of lycopene downstream flux caused by down-regulation of *LYCb *and *CCS *genes are critical for the formation of lycopene accumulation trait in sweet orange fruits.

## Methods

### Plant material and RNA preparation

The red-flesh mutant 'Hong Anliu' and the wild type 'Anliu' sweet orange (*C. sinensis *L. Osbeck), cultivated at the Institute of Citrus Research located in Guilin, Guangxi Province, China, were used as materials. The mutant 'Hong Anliu' sweet orange was a bud mutation from wild type 'Anliu' sweet orange; and they are with isogenic background as revealed from our previous molecular marker evaluations [27]. Sampling strategy is the same as previously published [32]. Fruit samples were harvested at 150, 170, 190, and 220 days after flowering (DAF) from three different trees, at each time point 10 representative fruits from each tree were collected. All samples were separated into peel and pulp, and immediately frozen in liquid nitrogen and kept at -80°C until use. Total RNA was extracted according to Liu et al. (2006) [54].

### Massively Parallel Signature Sequencing (MPSS)

The materials used for MPSS analyses were fruit pulps from mutant and wild type at stage 170 DAF (our previous results indicated that 170 DAF is the critical stage for transcriptional regulation; Figure 1). 20 μg of total RNA were sent to Beijing Genomics Institute (Shenzhen) where the libraries were produced and sequenced using Illumina's Genome Analyzer (Solexa). The MPSS were carried out essentially as previous studies [33,35], with some ideas lending from LongSAGE [55]. Briefly, the cDNA was digested with *Nla*III, and then ligated with the first adapter containing the recognition site of *Mme*I, a Type IIs endonuclease which cleave at sites 21 bp from the recognition site. After digestion by *Mme*I, the transcripts were ligated with the second adapter. With the sequencing primers designed based on the two adaptors, the sequence of the 21 bp representing each transcript can be determined via a series of enzymatic reactions on the microbeads. The derived reliable sequence was termed signature herein. The abundance of each signature is normalized to one million (transcripts per million, TPM) for the purpose of comparison between samples.

### Analyses of MPSS data

To remove signatures that may arise from errors in the MPSS procedure, two filters were applied to the derived signatures [34,35]. The first filter, the "reliability filter", was to remove low quality signatures containing ambiguous nucleotides or adaptor sequences. The second "significance" filter, with the intent to remove signatures that are consistently present at background levels, excluded signatures lower than 3 TPM in both libraries according to the criteria described by [34].

To link the expressed signatures to known genes from orange, the unigene dataset from TIGR gene index database http://compbio.dfci.harvard.edu/tgi/cgi-bin/tgi/gimain.pl?gudb=orange, 597 sweet orange transcription factors http://planttfdb.cbi.pku.edu.cn/web/index.php?sp=cs, and 964 sweet orange cDNA sequences from our lab were combined together as reference gene dataset. All the sequences were used to produce *in silico *signatures in both sense and antisense strands. The produced *in silico *signatures were stored in the reference signature database, through which the expressed MPSS signatures can be mapped on the corresponding EST contigs and singletons based on matches between MPSS signatures and *in silico *signatures, as described previously [38].

Significance level of the difference of signature frequency and transcript abundance between the two libraries was analyzed using Z-score method according to Kal et al. (1999) [41].

### Functional assignments of differentially expressed genes

To assign putative functions to differentially expressed genes between mutant and wild type, annot8r program was run locally to BLAST against a reference database that stores UniProt entries, their associated Gene Ontology (GO), Enzyme Commission (EC) and Kyoto Encyclopaedia of Genes and Genomes (KEGG) annotation [56]. The GO categorization results were expressed as three independent hierarchies for biological process, cellular component, and molecular function [57]. The biological interpretation of the differentially expressed genes was further completed by assigning to metabolic pathways using KEGG [58]. And for the identification of pathways significantly affected by the mutation, we focused on the metabolite pathways at least 3 genes affiliated.

### Real-time Quantitative RT-PCR (qRT-PCR) verification

Twenty five genes were chosen for confirmation by real-time quantitative RT-PCR. Primer pairs were designed with the Primer Express software (Applied Biosystems, Foster City, CA, USA). Primer sequences were presented in additional file 7. Quantitative Real-time PCR for gene expression analysis was performed on the ABI 7500 Real Time System (PE Applied Biosystems, Foster City, CA, USA) using *actin *gene as endogenous control according to Liu et al. (2007) [27]. Briefly, the primers for the target gene and *actin *were diluted in the SYBER GREEN PCR Master Mix (PE Applied Biosystems) and 20 μl of the reaction mix were added to each well. Reactions were performed by an initial incubation at 50°C for 2 min and at 95°C for 1 min, and then cycled at 95°C for 15 s and 60°C for 1 min for 40 cycles. Output data was generated by the instrument on-board software Sequence Detector Version 1.3.1 (PE Applied Biosystems).

### Chlorophyll content and Photosynthesis activity measurement

Chlorophyll extraction was performed according to van Schie et al. (2007) [59]. Briefly, 0.5 gram of ground leaf tissue was incubated with 5 ml of 95% ethanol at room temperature for 10 min. Extracts were cleared by centrifugation at 12 000 g for 1 min, then diluted 10 times with 95% ethanol. Three milliliters of the diluted extracts were measured using UV-1601 spectrophotometer (Shimadzu). Total chlorophyll content was caculated using the method of Lichtenthaler (1987) [60].

In situ rates of photosynthesis and stomatal conductance were measured with the TPS-1 portable photosynthesis system (PP Systems, Haverhill, MA). Three different trees in the field were used for each genotype; and four leaves on a plant were measured according to Hu et al. (2007) [61].

Statistical analyses (descriptive and *t*-test) were conducted using SPSS 10.0 software. Difference of compared sets were considered significant at *p *< 0.05.

## Authors' contributions

QX, KQY, ADZ, and JLY are responsible for generating the MPSS data and for interpretation of the results. KQY carried out qRT-PCR experiments. QX drafted the manuscript. QL and JCZ participated in research design and statistical analyses. XXD proposed and supervised the research. All authors read and approved the final manuscript.

## Supplementary Material

Additional file 1 **The saturation evaluations of the MPSS signatures in the libraries against the sequencing depth**. The results revealed that with the increase of total sequence number (sequencing depth), the number of new distinct signature decreased markedly; and particularly the newly appeared distinct signature with frequency >1 decreased to 0 when the total sequences reached 6 million, indicating enough information has been included in the MPSS data. Click here for file

Additional file 2 **The MPSS signature abundance distributions**. The abundance of each signature is calculated as a percentage of total signatures in the mutant (black column) and wild type (white column). Click here for file

Additional file 3 **Comparison of expression of each signature between the mutant and the wild type**. Four items are included, the first is the list of signatures with expression difference >2, the second is signatures differentially expressed at 0.05 significance level, the remained two are up- and down- regulated signatures at 0.05 significance level. Click here for file

Additional file 4 **Gene expression of sweet orange unigenes in the mutant**. This table listed genes expressed in sense and antisense strands and their expression level, also contains signature mapping information. Click here for file

Additional file 5 **Gene expression of unigenes in the wild type**. The table listed genes expressed in sense and antisense strands and their expression level, also contains signature mapping information. Click here for file

Additional file 6 **List of differentially expressed genes between the mutant and the wild type**. The table contained information of the differential expressed genes with expression difference >2, and genes differentially expressed at 0.05 significance level in sense and antisense strands. Click here for file

Additional file 7 Primers used for real-time quantitative RT-PCR for the verification of MPSS data. Click here for file

Additional file 8 **Real-time RT-PCR analyses of three genes involved in methylerythritol 4-phosphate (MEP) pathway which provided precursors of carotenoid biosynthesis**. Transcriptional expression of DXS (deoxyxylulose 5-phosphate synthase), HDS (hydroxymethylbutenyl 4-diphosphate synthase), HDR (hydroxymethylbutenyl 4-diphosphate reductase) were up-regulated in the mutant. Click here for file
