# Supplementary figures and images for: Comparative transcripts profiling reveals new insight into molecular processes regulating lycopene accumulation in a sweet orange (Citrus sinensis) red-flesh mutant
Source: BMC Genomics. 2009 Nov 18;10:540. doi: 10.1186/1471-2164-10-540 (PMC2784484; doi:10.1186/1471-2164-10-540)

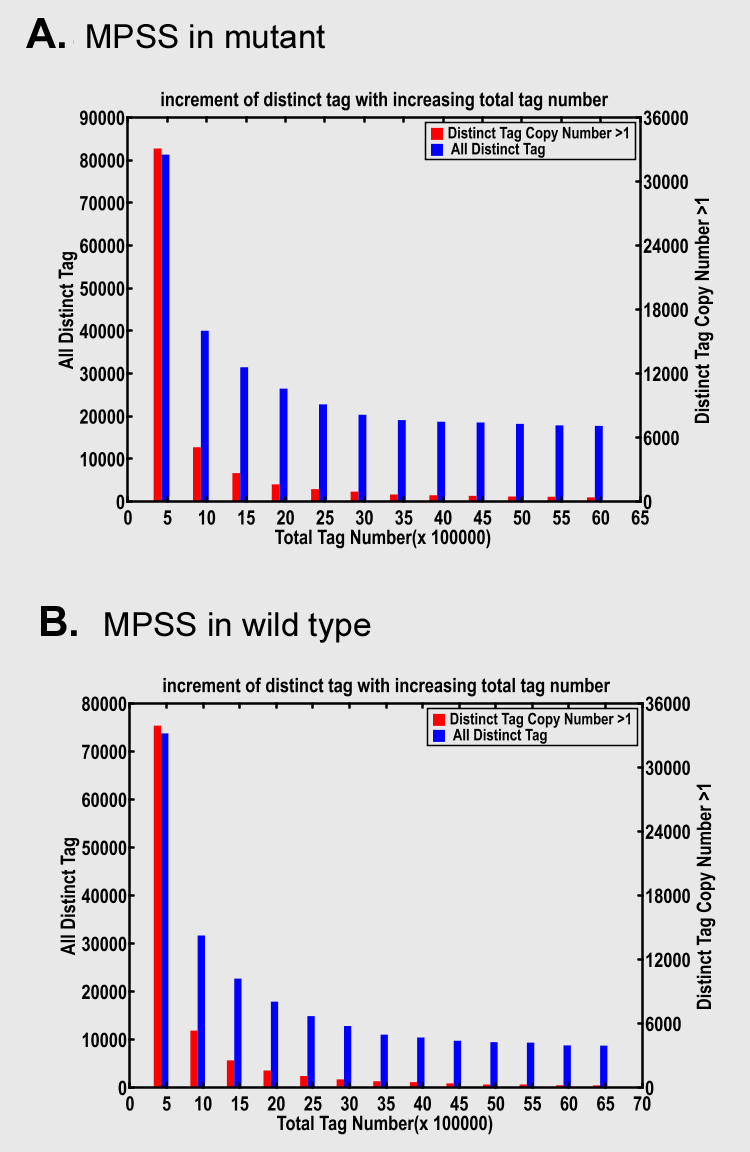

Supplement: Additional file 1 — The saturation evaluations of the MPSS signatures in the libraries against the sequencing depth. The results revealed that with the increase of total sequence number (sequencing depth), the number of new distinct signature decreased markedly; and particularly the newly appeared distinct signature with frequency >1 decreased to 0 when the total sequences reached 6 million, indicating enough information has been included in the MPSS data. [file 1471-2164-10-540-S1.TIFF]

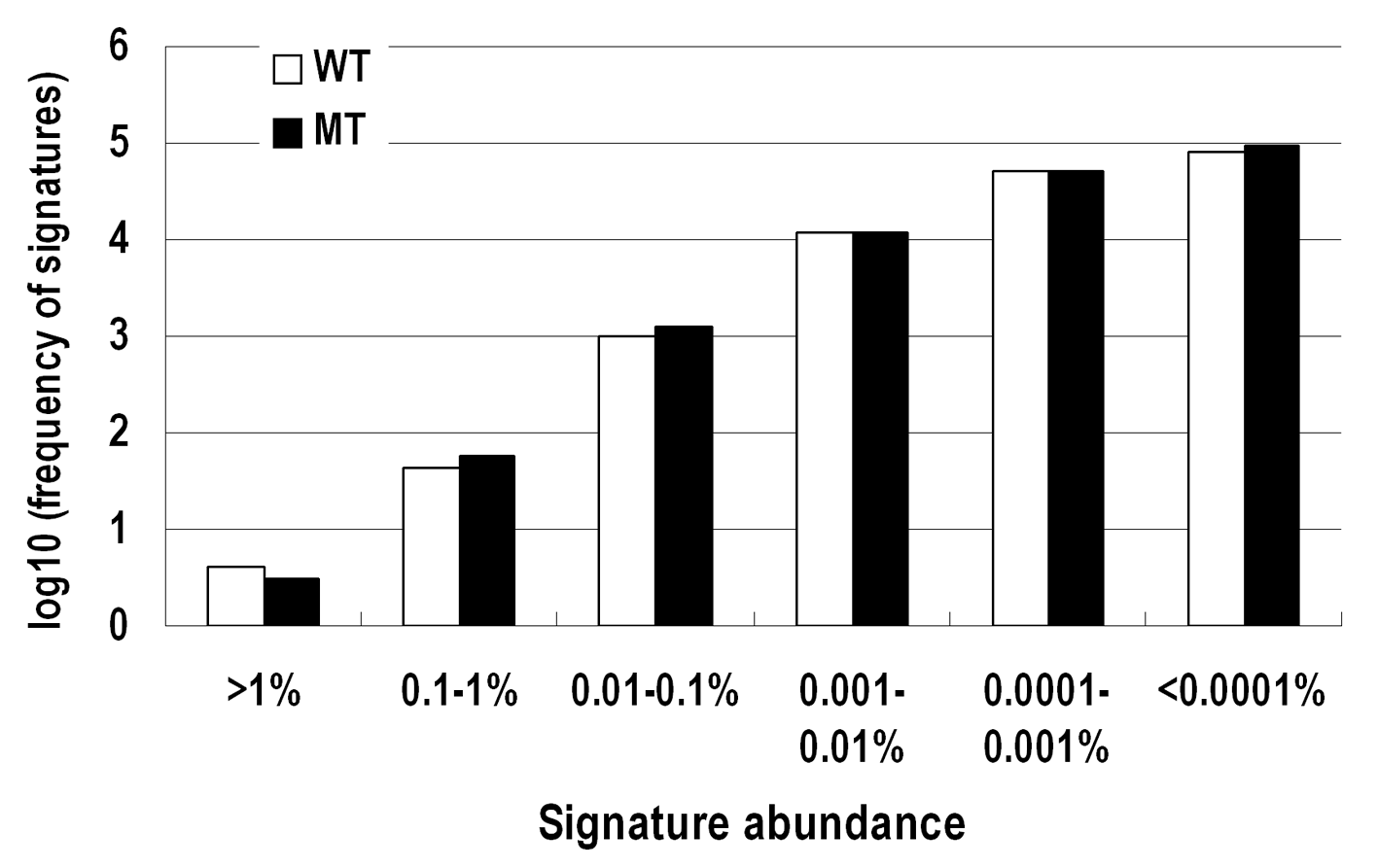

Supplement: Additional file 2 — The MPSS signature abundance distributions. The abundance of each signature is calculated as a percentage of total signatures in the mutant (black column) and wild type (white column). [file 1471-2164-10-540-S2.TIFF]

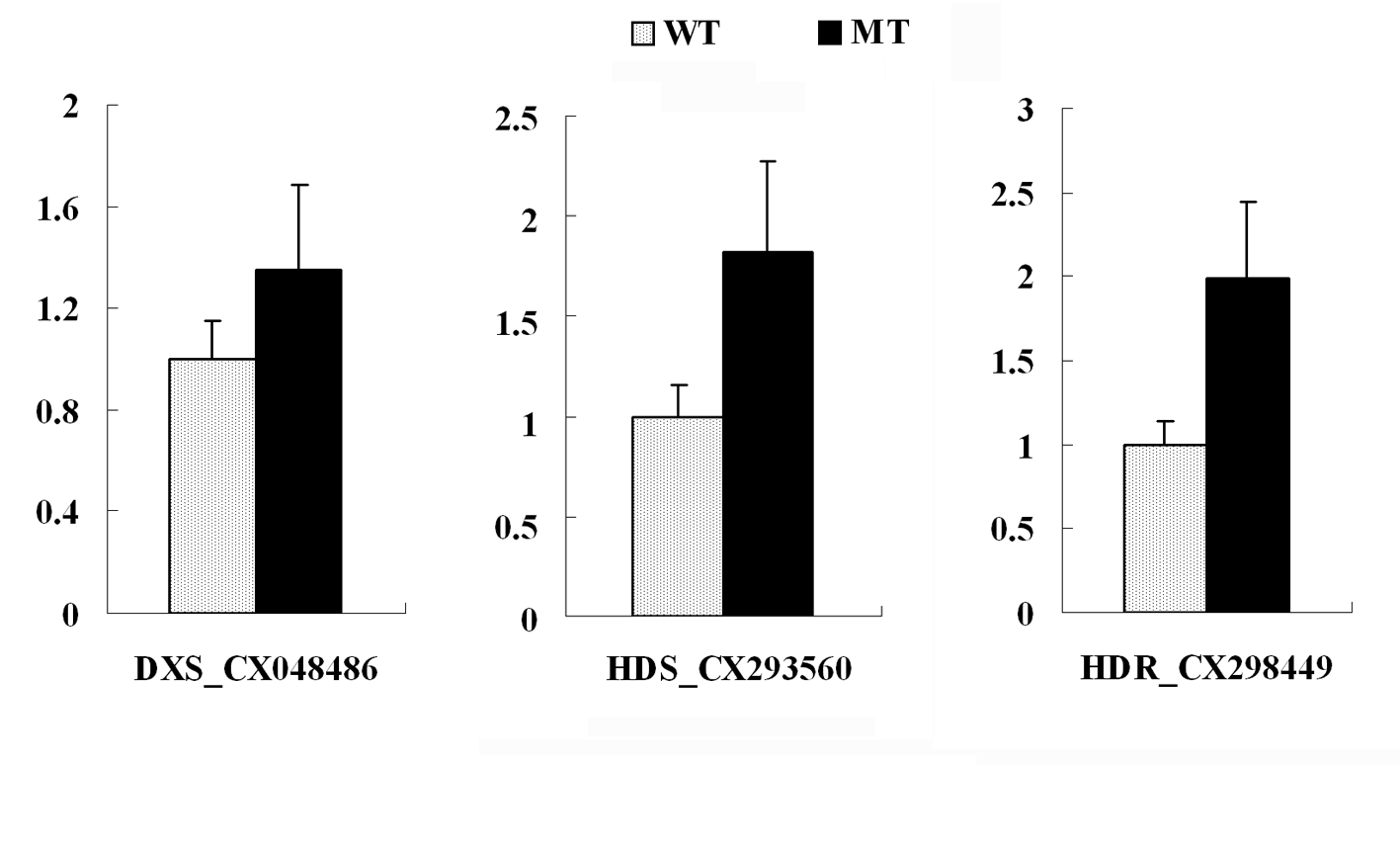

Supplement: Additional file 8 — Real-time RT-PCR analyses of three genes involved in methylerythritol 4-phosphate (MEP) pathway which provided precursors of carotenoid biosynthesis. Transcriptional expression of DXS (deoxyxylulose 5-phosphate synthase), HDS (hydroxymethylbutenyl 4-diphosphate synthase), HDR (hydroxymethylbutenyl 4-diphosphate reductase) were up-regulated in the mutant. [file 1471-2164-10-540-S8.TIFF]
